# Retrospective evaluation of vector-borne infections in dogs imported from the Mediterranean region and southeastern Europe (2007–2015)

**DOI:** 10.1186/s13071-018-3284-8

**Published:** 2019-01-11

**Authors:** Ingo Schäfer, Maria Volkmann, Pamela Beelitz, Roswitha Merle, Elisabeth Müller, Barbara Kohn

**Affiliations:** 1 Clinic for Small Animals, Faculty of Veterinary Medicine, Freie Universität Berlin, Berlin, Germany; 2 Institute of Veterinary Epidemiology and Biostatistics, Freie Universität Berlin, Berlin, Germany; 3 Chair for Experimental Parasitology, Faculty of Veterinary Medicine, Ludwig-Maximilians-Universität Munich, Munich, Germany; 4 Laboklin GmbH and Co.KG, Bad Kissingen, Germany

**Keywords:** Arthropod-transmitted infections, Vector-borne diseases, Laboratory diagnostics, Import

## Abstract

**Background:**

Canine vector-borne infections have gained importance in Germany due to growing tourist traffic and an increased import of dogs from abroad. Endemic regions for pathogens such as *Leishmania infantum*, *Hepatozoon canis*, *Ehrlichia canis*, *Anaplasma platys* and *Dirofilaria* spp. are the Mediterranean area and southeastern Europe. *Babesia* species and *Anaplasma phagocytophilum* are present all over Europe. The objective of this retrospective study was to evaluate the prevalence of vector-borne infections in dogs imported from defined endemic countries in the Mediterranean area and southeastern Europe.

**Methods:**

Medical records and laboratory test results of 345 dogs that were imported to Germany from 17 endemic countries and that were presented to the Small Animal Clinic at Freie Universität Berlin between 2007 and 2015 were retrospectively reviewed. A total of 1368 test results from external laboratories were descriptively analysed including 576 and 792 test results of direct and indirect detection methods, respectively.

**Results:**

Overall, 35% (122/345 dogs) were positive for at least one pathogen. Concurrent infections with two to four pathogens were detected in 8% of the dogs (27/345). The positive results were: *L. infantum* 21% (66/314 dogs; methods: PCR 20/79, IFAT or ELISA 63/308 dogs), *E. canis* 16% (45/278 dogs; methods: PCR 8/68, IFAT 43/257 dogs), *H. canis* 11% (3/28 dogs; method: PCR), *Babesia* spp. 10% (25/251 dogs; methods: *Babesia* spp. PCR 3/98, *B. canis*/*vogeli* IFAT or ELISA 22/214 and *B. gibsoni* IFAT 0/13 dogs), *Dirofilaria* spp. 7% (13/178 dogs; methods: *D. immitis* Ag-ELISA 8/156, Knott’s test 7/95, microfilariae PCR 5/23 dogs) and *A. platys* 5% (1/21 dogs; method: PCR). None of 8 tested dogs were positive in a combined *Babesia* spp./*Hepatozoon* spp. PCR test.

**Conclusions:**

Dogs, which are imported from countries which are endemic for vector-borne infections should be thoroughly tested using direct and indirect detection methods. Potential owners of imported dogs should be informed about the diseases, risks and incubation periods.

## Background

Blood-feeding arthropods transmit parasitical, bacterial or viral pathogens, which can result in infections in a host. With respect to long incubation times in the case of some infections and the wide range of unspecific clinical signs, especially for dogs with multiple infections, diagnosis and therapy might be difficult [1–4]. The occurrence of these so-called vector-borne infections depends on the geographical existence of the vectors and reservoirs [5]. The import of infected dogs has several effects in non-endemic countries. Pathogens can be imported to non-endemic countries *via* infected dogs. Non-endemic vectors can be imported and gain vector competence. Endemic vectors can be infected with non-endemic pathogens and may serve as alternate competent vectors by blood-feeding on a naive dog. Due to the import of infected dogs and climatic changes in Europe, vectors have the potential to infest non-endemic environments in more northern countries such as Germany and spread pathogens in accordance with the vectors’ competence [1, 6]. Areas in Europe endemic for pathogens such as *Leishmania* spp., *Hepatozoon canis*, *Ehrlichia canis*, *Anaplasma platys* and *Dirofilaria immitis* are the Mediterranean region and southeastern Europe. Meanwhile *Anaplasma phagocytophilum* and *Babesia* spp. are endemic in Germany [7]. *B. canis* has been ascertained sporadically in certain regions in Germany [8–12], including the area Berlin-Brandenburg [13]. Two cases of autochthonous infections with *B. gibsoni* have been previously described in Germany [14] and occasional autochthonous *Dirofilaria repens* infections in Germany have been detected [15–17].

Dogs in endemic countries are at high risk of vector-borne infections. Only a few studies have described test results for vector-borne infections in dogs imported from endemic countries to Germany [18–23]. Therefore, the objective of the present study was to evaluate the prevalence of vector-borne infections in a population of dogs that were imported from endemic regions in the Mediterranean area and southeastern Europe, and that were presented to the Small Animal Clinic at Freie Universität (FU) Berlin, Germany.

## Methods

This study was performed retrospectively. The dogs were presented to the Small Animal Clinic at FU Berlin between January 2007 and December 2015 and were identified by keyword search in the clinic’s software program and enquiries to external laboratories (Laboklin, Bad Kissingen; Institute for Experimental Parasitology, Ludwig-Maximillians-University, Munich). Only dogs with an origin from a defined endemic country (13 Mediterranean countries and 4 countries in southeastern Europe) and at least one direct or indirect examination for vector-borne infections were included in the study (Tables 1 and 2).

**Table 1:** Direct and indirect methods of detection for vector-borne infections initiated in imported dogs

Infectious agent	Test	LMU Munich	Laboklin
*Ehrlichia canis*	PCR	Applied Biosystems TaqMan© Real Time PCR [71]	TaqMan© Real Time PCR (in-house test)
	Ab-IFAT	MegaScreen© FLUOEHRLICHIA canis (MegaCor Diagnostik GmbH, Hörbranz, Austria; ≥ 1:40 positive)	MegaFLUO© EHRLICHIA canis (MegaCor Diagnostik GmbH, Hörbranz, Austria; ≥ 1:80 positive)
*Anaplasma platys*	PCR	Applied Biosystems TaqMan© Real Time PCR [72]^a^	TaqMan© Real Time PCR (in-house test)
*Leishmania infantum*	PCR	Applied Biosystems TaqMan© Real Time PCR [73]	TaqMan© Real Time PCR [74]
	Ab-IFAT	*Leishmania infantum* MON-1 [75]; ≥ 1:64 positive	MegaFLUO© LEISH (MegaCor Diagnostik GmbH, Hörbranz, Austria; › 1:64 positive)
	Ab-ELISA	-	Civtest© Canis Leishmania (Hipra, Amer, Spain; › 1,1 LE positive)
*Babesia* spp.	PCR^b^	PCR (*18S* rRNA) with gel electrophoresis [76]^c^	PCR (*18S* rRNA) with gel electrophoresis [77]^d^
*Babesia canis* ^e^	Ab-IFAT	MegaScreen© FLUOBABESIA canis (MegaCor GmbH, Hörbranz, Austria; ≥ 1:64 positive)	MegaFLUO© BABESIA canis (MegaCor GmbH, Hörbranz, Austria; ≥ 1:40 positive)
	Ab-ELISA	–	Babesia ELISA Dog (Afosa, Blankenfelde-Mahlow, Germany; 19 TE positive)
*Babesia gibsoni*	Ab-IFAT	MegaScreen© FLUOBABESIA gibsoni-Testkit (MegaCor GmbH, Hörbranz, Austria; ≥ 1:64 positive)	MegaFLUO© BABESIA gibsoni (MegaCor GmbH, Hörbranz, Austria; ≥ 1:32 positive)
*Babesia* spp./*Hepatozoon* spp.	PCR^b^	In-house protocol	–
*Hepatozoon canis*	PCR	PCR (*18S* rRNA) with gel electrophoresis [78]^f^	TaqMan© Real Time PCR (in-house test)
*Dirofilaria* spp.	Knottʼs test	Modified Knottʼs test [79]	Modified Knottʼs test [79]
Microfilariae	PCR	PCR (IST-2) with gel electrophoresis [80]^c^	TaqMan© Real Time PCR (in-house test)^f^
*Dirofilaria immitis*	Ag-ELISA	Dirochek© Canine Heartworm Antigen Test Kit (Synbiotics Corporation, San Diego, California 92127, US Veterinary License No. 312; Megacor)	FASTest© HW Antigen (MegaCor GmbH, Hörbranz, Austria)

^a^In combination with *A. phagocytophilum* PCR due to sequence homology

^b^Differentiation between different species possible by request of veterinarian

^c^Species differentiation after sequencing of the PCR product and comparison with the database GenBank (NCBI Blast Search)

^d^Sequencing of the PCR-product by request of the veterinarian

^e^Serological cross-reactions between *B. canis* und *B. vogeli* possible

^f^*18S* rRNA, 2012–2015 (2007–2012 no data available)

*Abbreviations*: LMU Munich, Institute for Experimental Parasitology, Ludwig-Maximilians-University Munich, Germany; Laboklin, Laboklin, Bad Kissingen, Germany; PCR, polymerase chain reaction; Ag-ELISA, antigen enzyme-linked immunosorbant assay; Ab-IFAT, immunofluorescence antibody test; Ab-ELISA, antibody enzyme-linked immunosorbant assay

**Table 2:** Number of vector-borne infections in dogs import from endemic countries (number of monoinfections/number of multiple infections)

Country of origin	No. of dogs tested positive/total (%)	*E. can*	*A. pla*	*L. inf*	*B.* spp^a^	*B. can* ^b^	*H. can*	*D.* spp^c^	*D. imm*	*D. rep*	*Ac. rec*
Spain	67/186 (36)	10/6	1/–	35/14	–/–	4/8	–/–	–/1	2/2	–/1	–/1
Greece	22/48 (46)	8/5	–/–	7/3	–/–	–/4	–/–	–/–	1/1	–/1	–/–
Hungary	4/19 (21)	–/–	–/–	–/–	1/–	1/–	–/–	–/–	1/–	1/–	–/–
Italy	3/19 (16)	–/1	–/–	1/–	–/–	–/1	–/–	–/–	1/–	–/–	–/–
Portugal	6/12 (50)	3/–	–/–	1/–	–/–	2/–	–/–	–/–	–/–	–/–	–/–
Bulgaria	4/9 (44)	3/–	–/–	–/–	–/–	–/–	1/–	–/–	–/–	–/–	–/–
France	0/9	–/–	–/–	–/–	–/–	–/–	–/–	–/–	–/–	–/–	–/–
Croatia	4/8 (50)	2/–	–/–	–/–	–/–	1/–	1/–	–/–	–/–	–/–	–/–
Turkey	1/8 (13)	1/–	–/–	–/–	–/–	–/–	–/–	–/–	–/–	–/–	–/–
Cypress	4/7 (57)	1/2	–/–	1/–	–/–	–/1	–/1	–/–	–/–	–/–	–/–
Malta	4/7 (57)	1/1	–/–	–/3	–/–	–/2	–/–	–/–	–/–	–/–	–/–
Romania	1/7 (14)	1/–	–/–	–/–	–/–	–/–	–/–	–/–	–/–	–/–	–/–
Slovenia	0/3	–/–	–/–	–/–	–/–	–/–	–/–	–/–	–/–	–/–	–/–
Israel	1/1 (100)	1/–	–/–	–/–	–/–	–/–	–/–	–/–	–/–	–/–	–/–
Montenegro	1/1 (100)	–/–	–/–	1/–	–/–	–/–	–/–	–/–	–/–	–/–	–/–
Tunisia	0/1	–/–	–/–	–/–	–/–	–/–	–/–	–/–	–/–	–/–	–/–
Total	122/345 (35)	31/15	1/–	46/20	1/–	8/16	2/1	0/1	5/3	1/2	–/1

^a^Not differentiated *Babesia* spp. PCR (polymerase chain reaction)

^b^Serological cross-reactions between *B. canis* and *B. vogeli* possible

^c^Non-differentiated Knott’s test

*Abbreviations*: *E. can*, *Ehrlichia canis*; *A. pla*, *Anaplasma platys*; *L. inf*, *Leishmania infantum*; *B.* spp., *Babesia* spp.; *B. can*, *Babesia canis*; *H. can*, *Hepatozoon canis*; *D.* spp., *Dirofilaria* spp.; *D. imm*, *Dirofilaria immitis*; *D. rep*, *Dirofilaria repens*; *Ac. rec, Acanthocheilonema reconditum*

Direct testing methods included PCR, Ag-ELISA and Knott’s test. Indirect testing methods included IFAT and Ab-ELISA (Table 1). Descriptive statistical analysis was ascertained *via* SPSS for Windows (version 24.0, SPSS Inc., Armonk, NY, USA). Chi-square test was used to compare categorical variables and results are given as percentages. Statistical significance was set at *P* < 0.05.

## Results

### Signalment/history

In total 345 dogs were imported from 16 endemic countries (Table 2); no dogs were brought to Germany from Serbia. Most dogs originated from Spain (186/345, 54%), Greece (48/345, 14%), Italy (19/345, 6%), Hungary (19/345, 6%) and Portugal (12/345, 3%). Information on sex and breed was available for 344 dogs: 179 (52%) were females and 165 (48%) were males; 202 (65%) were mixed breed and 122 (35%) were purebred dogs, belonging to 59 different breeds. The age was known in 335 dogs, with a median of 4.7 (0.2–16.1) years. A total of 287/345 cases (83%) were presented with clinical signs and the remainder without clinical signs were presented for routine medical check-up. The time between import to Germany and presentation in the clinic is depicted in Table 3. Clinical signs were present in 41/50 dogs (82%) living in Germany for 0–2 months, 28/33 (85%) living in Germany for 2–6 months, 40/44 (91%) living in Germany for 6–12 months, 81/98 (83%) living in Germany for 1–5 years, 16/18 (89%) living in Germany for 5–7 years and 24/35 (69%) living in Germany for longer than 7 years.

**Table 3:** Number of dogs tested positive for vector-borne infections after time between import and presentation in the clinic

Period	Positive/total (%)	*E. canis*	*A. platys*	*L. infantum*	*Babesia* spp.	*H. canis*	*Dirofilaria* spp.	Multiple infections
No data	22/67 (33)	6	–	8	4	1	1	2
0–2 months	26/50 (52)	8	1	6	2	1	1	7
2–6 months	10/33 (30)	2	–	1	1	–	1	5
6–12 months	16/44 (36)	3	–	5	1	–	1	6
1–5 years	39/98 (40)	8	–	22	1	–	2	6
5–7 years	6/18 (33)	3	–	3	–	–	–	–
> 7 years	3/35 (9)	1	–	1	–	–	–	1
Total	122/345 (35)	31	1	46	9	2	6	27

### Laboratory diagnostics

In total, 1368 tests for vector-borne infections were initiated between January 2007 and December 2015. Thereof 55/576 direct (10%) and 128/792 indirect tests (16%) were positive (Table 4). Twenty-five of 251 dogs (10%) were positive for *Babesia* spp.: in two of these dogs, *B. canis* was identified after species differentiation using PCR; in one dog (PCR positive) and in 22 serologically positive dogs, species differentiation was not performed. Thirteen of 178 dogs (7%) were positive for microfilariae. In eight of 13 dogs *D. immitis*, three of 13 dogs *D. repens* and one of 13 dogs, *Acanthocheilonema reconditum* was detected. In one case further microfilarial differentiation was not performed.

**Table 4:** Number of positive tests for vector-borne infections in dogs imported to Germany

Infectious agent/test	No. of dogs tested positive/total (%)	Direct tests (positive/total)	Indirect tests (positive/total)
*Ehrlichia canis*	45/278 (16)	8/68^a^	43/257^b^
*Anaplasma platys*	1/21 (5)	1/21^a^	–
*Leishmania infantum*	66/314 (21)	20/79^a^	57/276^b^; 6/32^c^
*Babesia* spp.	3/98 (3)	3/98^a,d^	–
*Babesia canis* ^e^	22/213 (10)	–	20/187^b^; 2/27^c^
*Babesia gibsoni*	0/13 (0)	–	0/13^b^
*Hepatozoon canis*	3/28 (11)	3/28^a^	–
*Babesia* spp./*Hepatozoon* spp.	0/8 (0)	0/8^a^	–
*Dirofilaria immitis*	8/156 (5)	8/156^f^	–
Microfilariae	5/23 (22)	5/23^a^	–
Modified Knott’s test	7/95 (7)	7/95	–
Total	122/345 (35)	55/576 (10%)	128/792 (16%)

^a^Polymerase chain reaction

^b^Immunofluorescence antibody test

^c^Antibody enzyme-linked immunosorbant assay

^d^2/3 positive PCR-tests were differentiated as *B. canis*, 1/3 was not differentiated

^e^Serological cross-reactions between *B. canis* und *B. vogeli* possible

^f^Antigen enzyme-linked immunosorbant assay

Twenty-seven of 345 dogs (8%) were infected with two to four pathogens. In 24/345 dogs two pathogens were detected: nine dogs with *Babesia* spp. and *L. infantum* (seven dogs from Spain, two dogs from Malta), six dogs with *E. canis* and *L. infantum* (three dogs from Spain, two dogs from Greece, one dog from Malta), four dogs with *E. canis* and *Babesia* spp. (two dogs from Greece, one dog each from Italy and Cypress) and in one dog each the following co-infections were detected: *L. infantum* + *D. repens* (Spain); *L. infantum* and positive Knott’s test (*Dirofilaria* spp. not differentiated, Spain); *E. canis* + *Acanthocheilonema reconditum* (Spain); *L. infantum* + *D. immitis* (Spain); and *E. canis* + *H. canis* (Cypress). Two dogs imported from Greece were positive for three pathogens, in one *E. canis* + *Babesia* spp. + *D. repens* and in the other *Babesia* spp. + *L. infantum* + *D. immitis*. In one dog from Spain the following four pathogens were detected: *E. canis*, *Babesia* spp., *L. infantum* and *D. immitis*.

**Fig. 1:**
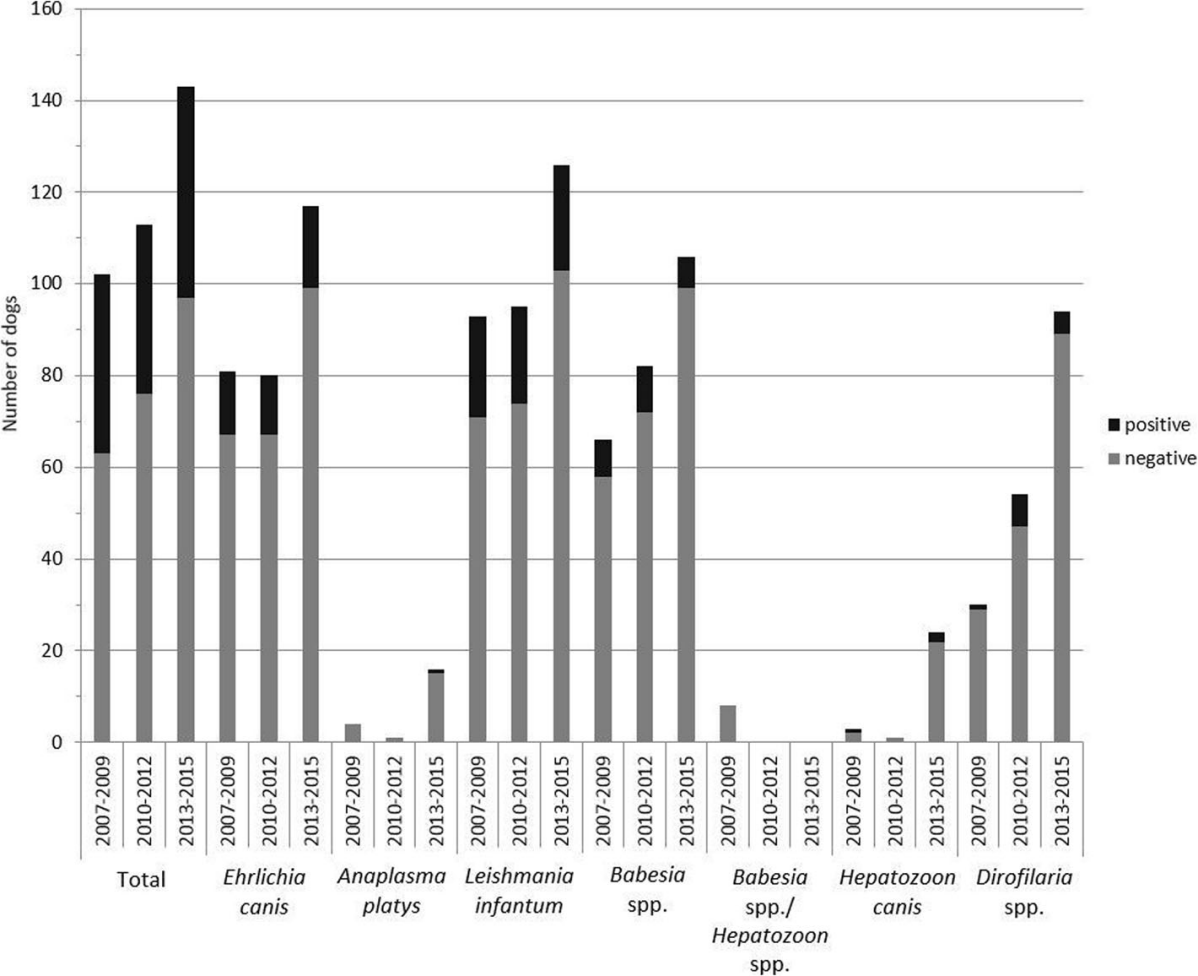
Number of dogs tested for vector-borne infections between 2007 and 2015

The number of dogs tested for vector-borne infections was the highest in the period 2013–2015 compared to the periods 2007–2009 and 2010–2012 (Fig. 1). Tests using a combined PCR for *Babesia* spp*.*/*Hepatozoon* spp*.* were only initiated in the year 2008. The number of dogs with positive test results for vector-borne infections (Fig. 1) was not significantly different between the three time periods (2007–2009, 2010–2012, 2013–2015), neither for total analyses (*χ*^2^ = 0.925; *df* = 2; *P* = 0.630) nor for *E. canis* (*χ*^2^ = 0.107; *df* = 2; *P* = 0.948), *L. infantum* (*χ*^2^ = 0.144; *df* = 2; *P* = 0.931), *Babesia* spp. (*χ*^2^ = 1.954; *df* = 2; *P* = 0.376) and *Dirofilaria* spp. (*χ*^2^ = 3.953; *df* = 2; *P* = 0.139). No statistical analysis was performed for *A. platys* and *H. canis* because a minimum of ten dogs should be tested for every pathogen in every period. In proportion to the total number of dogs presented in the clinic between 2007 and 2015 the percentage of dogs tested for vector-borne infections was 1% (345/33925 dogs). In 2007 the proportion was the highest with 1.2% (37/3110 dogs). In the periods 2008–2009 and 2011–2015 the proportion ranged between 0.6 and 0.9%. The proportion was lowest in the year 2010 with 0.4% (28/6537 dogs).

## Discussion

In 35% of 345 imported dogs tested for vector-borne infections, at least one pathogen was detected. The most common pathogen was *L. infantum* with 21% of tested dogs being positive, followed by *E. canis* with 16%. Eleven percent of dogs were positive for *H. canis* and 10% for *Babesia* spp. *Anaplasma platys* was detected in 5% of tested dogs. Eight percent of dogs were positive for multiple pathogens. Only dogs originating from the Mediterranean region had positive test results for more than one pathogen, especially *E. canis* and *Babesia* spp. Both pathogens can induce immunosuppression which can promote an infection with further pathogens [24, 25]. The prevalence of vector-borne infections is, amongst other biotic and abiotic factors, determined by the presence of competent vectors. *B. vogeli*, *E. canis*, *A. platys* and *H. canis* are reliant on *Rhipicephalus sanguineus* as a vector, which can transmit various individual pathogens and thus more than one infection [2]. As *R. sanguineus* can only temporarily survive as an outdoor tick in temperate regions including Germany and as an indoor population only in year-round tempered buildings [26], the exposure reported here can most likely be attributed to previous infections in the dogs’ country of origin. Certain *Dirofilaria* species could develop natural transmission cycles in Germany and to date this has been proven for *D. repens* [27]. As *D. immitis* and *A. reconditum* are not endemic in Germany, infections with these parasites are most likely imported. Regarding the three dogs (from Spain, Hungary, Greece) that were infected with *D. repens* and presented between 2011 and 2014, an infection would have been possible in their home country as well as (though perhaps less likely) in the region Berlin-Brandenburg. Studies conducted in Brandenburg showed that climatic conditions in this region do allow the development to the infectious L3 larva during limited periods and certain temperature frames [17, 28]. The pathogen was detected in a local mosquito population in Brandenburg in 2011 and 2012 [29].

In total, 25 dogs were infected with *Babesia* spp. in our study. Autochthonous infections with *B. canis* in certain regions within Germany, such as the Upper Rhine [10], Bavaria [9, 30], Lower Saxony [31], Rhineland-Palatinate [12] and Brandenburg [13, 32], have been described. In a questionnaire-based survey with 313 *Babesia-*infected German dogs which had never left the home country, autochthonous infections have been found in dogs from Saarland (number of positive dogs = 225), Baden-Württemberg (*n* = 20), Bavaria (*n* = 18), North Rhine-Westphalia (*n* = 18), Rhineland Palatinate (*n* = 6), Thuringia (*n* = 5), Saxony (*n* = 4), Saxony-Anhalt (*n* = 4), Hesse (*n* = 4), Lower Saxony (*n* = 3), Schleswig Holstein (*n* = 3), Berlin (*n* = 2) and Brandenburg (*n* = 1) [11]. Twenty-two of the 25 dogs in our study were only serologically positive for *Babesia* spp., with no differentiation between an infection with *B. vogeli* or *B. canis*. Twenty-one of 22 dogs originated from the Mediterranean area (mainly Spain and Greece), and one of the 22 dogs was originally from Hungary. Generally, *B. canis* occurs more often in central Europe, but it has also been found in the Mediterranean [33]. An infection with *B. canis* in Germany would be possible, but since most of the 22 serologically positive dogs did not show clinical signs of acute babesiosis (*n* = 18), were imported within one to seven weeks (*n* = 3) and/or were PCR-negative (*n* = 9), an infection within the country of origin seems more likely. Three of 25 *Babesia-*positive dogs had a positive PCR result. One dog with hemolytic anemia was from Hungary, but its PCR result was not further differentiated. This dog had only been in Germany for four weeks and was not serologically tested; an infection with *B. canis* in Hungary, which is an endemic region for this pathogen, was assumed. Two dogs were originally from Spain, and further differentiation of the PCR results revealed *B. canis*. These two dogs from Spain were presented due to hemolytic anemia and masticatory muscle myositis in 2010 and 2011, in which two studies did not detect *B. canis* in *Dermacentor reticulatus* ticks from Berlin-Brandenburg [34, 35]. Recently, in 2015, four dogs with *B. canis* infection were described, which were most likely infected in Berlin-Brandenburg [32]. Therefore, the infection of these two dogs from Spain could have occurred in Berlin-Brandenburg or in their country of origin. Ten of 22 dogs tested serologically positive for *Babesia* spp. had co-infections with *L. infantum*, which implies the possibility of serological cross-reactions between the two pathogens.

For *B. gibsoni*, vertical infections [36], as well as infections *via* bite wounds, saliva and blood contact [37–39], have to be considered as a transmission route, especially in non-endemic regions for specific vectors [40]. As *B. gibsoni* infections are usually of low importance and low prevalence in Germany, an infection occurring in the endemic country of the vector seems more likely for the dogs in our study.

Regarding *L. infantum*, individual cases of infections transmitted *via* mating [41, 42], transplacental [43–46] and bite wounds [47] have been described. It is most likely that these routes of infection do not play a part in our analysis.

In comparison to previous studies by Röhrig et al. [18] and Menn et al. [20], the amount of positive tested dogs was similar (Table 5). In addition to the Mediterranean area and southeastern Europe, regions such as northern Europe and Russia were considered as endemic regions in two studies [18, 20], respectively. However, some pathogens are not endemic in these regions, which could explain the lower prevalence of vector-borne infections in these studies. Furthermore, comparisons between the studies were difficult because of discrepancies regarding the spectrum of vector-borne infections being analysed. The inclusion of *A. phagocytophilum* with high seroprevalence had an influence on the total prevalence of vector-borne infections [18, 20]. Excluding the pathogen *A. phagocytophilum* from analyses, *L. infantum*, *E. canis* and *B. canis* were the most common infections (Table 5), which coincides with our results. The percentage of infections with *Dirofilaria* spp. was higher in our study (7%) than in the study implemented by Röhrig et al. [18] (3%).

**Table 5:** Prevalences of vector-borne infections in selected retrospective studies in imported dogs in Germany (positive results/number of tested dogs)

Infectious agent	Detection methods	Röhrig et al. [18]^a^	Menn et al. [20]^b^
Period		2004–2008	2004–2009
*Ehrlichia canis*	Direct	5.3 (3/57)	–
	Indirect	10.8 (299/2763)	10.1 (492/4308)
*Hepatozoon canis*	Direct	1.1 (26/2289)	2.2 (133/4548)
*Anaplasma phagocytophilum*	Direct	5.0 (9/179)	–
	Indirect	29.8 (130/436)	22.4 (332/1481)
*Babesia canis*	Direct	0.5 (5/2289)	–
	Indirect	8.9 (251/2819)	24.3 (1138/3507)
*Leishmania infantum*	Direct	14.9 (14/94)	–
	Indirect	9.6 (292/3049)	12.2 (569/3682)
*Dirofilaria immitis*	Direct	3 (68/2223)	–
Knott’s test	Direct	6.4 (108/1685)	7.7 (372/4309)
Prevalence	Positive dogs	– (–/3531)	43.7 (2044/4681)

^a^Imported dogs (94% from Mediterranean countries in Europe)

^b^Proportion of dogs with holiday stays abroad (*n* = 87, 1.8%) and number of dogs without anamnesis (*n* = 368, 7.9%)

As in our study, the above-mentioned studies did not test all pathogens *via* direct and indirect detection methods. In one of the studies 5.5% of direct and 20.5% of indirect testing methods were positive [18]. In all publications, the number of positive results tested *via* direct detection methods was considerably lower than those detected by indirect testing methods. In our study 10% of the direct test results and 16% of the indirect test results were positive. This implies that the infection was not acute in most dogs.

The number of multiple infections varied in the literature between 2.6% [18] and 15% [20]. Our results fall between these described prevalences. In the study by Menn et al. [20] import history was available in 4226 out of 4681 dogs (90.3%). The remainder either accompanied their owners abroad or anamnestic information was non-existent. Dogs accompanying their owners on travels have a lower risk of vector-borne infections than imported dogs [6, 19, 48, 49]. A prospective study examined dogs before starting their journey and at different time points after returning. A lower risk of infection for the individual dog was noticed for temporary visits in endemic countries [50].

For diagnostic purposes, it is important to differentiate between exposure to a pathogen, infection with a pathogen and clinical disease caused by an infection. Direct testing methods detect an antigen and might be positive if an infection is suspected to have occurred recently and no seroconversion has occurred yet [51]. PCR testing is also recommended in puppies, due to the existence of maternal antibodies [51]. In direct detection methods, an adequate amount of antigen has to be present in the bloodstream for a positive result, meaning that a negative result does not exclude the existence of an infection. A dog tested positive by direct testing methods can be classified as infected. Indirect testing methods detect antibodies against a pathogen. It is not possible to differentiate between exposure and infection with a single test. In the case of a four-fold rise or fall in titres, an infection is likely. On one hand indirect detection methods like IFAT and ELISA have a high sensitivity and specificity [52], but on the other hand limitations of serological examinations are cross-reactions, false-negative results in young or immunosuppressed dogs, and the premature implementation of tests post-infection before the beginning of seroconversion. In IFAT the subjective awareness, especially in borderline titre values, plays an important role and has effects on sensitivity and specificity [53]. Therefore, a combination of indirect and direct detection methods is recommended whilst taking the prepatency of the individual pathogen into account, especially in imported dogs with an unknown time of infection. Important information includes the dog’s country of origin, the time of import to Germany, domestic and international travels and clinical signs. Following this, direct and/or indirect detection methods for the particular pathogen should be initiated. A differentiation between exposure/infection and clinical disease should be made on the basis of clinical and clinicopathological signs and by exclusion of differential diagnoses causing similar signs.

In *Dirofilaria*, the prepatency of six months must be considered. In 71 dogs of the study, which were presented within the first six months after import, there was the possibility of a false-negative result due to the premature initiation of tests. Microfilariae can survive in the bloodstream for two years, which means that dogs treated with adulticide medication or dogs with naturally eliminated infections are positive for microfilaria but negative when tested for antigens using ELISA during this time. This was the case for one dog of the study. In dogs treated prophylactically, the antigen release can be delayed for up to nine months post-infection [54]. A negative result for microfilariae with a positive proof of antigen, as seen in two dogs in the study, can occur for several reasons: the prepatency of six months post-infection, infection with same-sex worms, medicinal sterilisation of adult worms by use of macrolides and/or doxycycline, previous treatment against microfilariae or immune-mediated elimination of the circulating microfilaria in the blood [55]. Due to the necessity of detecting all *Dirofilaria* stages, an examination *via* an enrichment process for microfilariae (Knott’s test) or microfilariae PCR combined with an antigen test is recommended. The sensitivity of PCR for the detection of *L. infantum* depends on the number of parasites in the examined medium [56]. In one study, sensitivities of 87% in blood and 100% in bone marrow are described [57]. Infected dogs often show low or borderline antibody titres because of the dominating TH1-immune response [58]. Seroconversion after natural infection may occur at different times according to literature: one to three months post-infection [59], 12 months post-infection [60] and up to 12–36 months post-infection [61]. The possibility of an absent seroconversion in infected dogs is also discussed [61]. Tests for *Leishmania* and *Dirofilaria* should be repeated after six months if the initial result is negative because of the long time for seroconversion of *Leishmania* and the long prepatency for *Dirofilaria* [51]. For these pathogens in particular, there is the possibility of a higher number of infections than stated in our study.

*Ehrlichia canis* can be detected *via* PCR before the beginning of seroconversion, between days four and ten post-infection [62, 63] and *via* IFAT starting at day 14 post-infection (range one to four weeks) [62–64]. Due to the early seroconversion of this pathogen, the risk for false-negative results (unlike for *Leishmania* and *Dirofilaria*) on the grounds of premature initiation of tests is low. PCR is considered to be the most sensitive method of detection for *A. platys* [65]. *Babesia canis* can be detected in blood seven days post-infection *via* PCR [66]. Specific antibodies for *B. canis* were detected 14 days post-infection in experimentally infected dogs. *Babesia canis*, *B. vogeli* and *B. rossi* can cross-react in an IFAT or ELISA. On a species-level, *Babesia* spp. can also cause cross-reactions in an IFAT as well as in an antibody ELISA when whole antigen is used, for example between *B. canis* and *B. gibsoni* [33]. The serological detection of *H. canis* is not common in routine diagnosis and PCR is considered to be the best method of detection [67, 68]. In our study, 28 dogs were tested for *H. canis*, with a greater number undergoing tests between 2013 and 2015. This shows that there is an increasing awareness for this vector-borne infection. Immunosuppressed, immunodeficient and co-infected dogs, in particular, suffer from *H. canis* [69]. In our study one third of *H. canis* positive tested dogs suffered from further infections.

This survey included examinations in clinically sick as well as asymptomatic dogs. The prevalence for vector-borne infections also depends on the health status of the tested dogs [70]. Prophylaxis is especially important for dogs accompanying their owners during travels. In a literature review, great regional differences in prevalence within various endemic countries were presented [70]. Regarding the risk of infection, there are not only differences between the countries, but also between the individual regions within a country. Our study retrospectively included the countries of origin, but not the different regions within these individual countries.

The evaluability of the results was limited due to the retrospective character of the study and the fact that not all tests were performed in all dogs. Reasons for this could be that owners were financially restricted, tests had already been initiated beforehand, or invalid test results. Additionally, the precision of diagnostic testing methods improved between 2007 and 2015. Nevertheless, the amount of 122/345 (35%) imported dogs being tested positive for vector-borne infections is remarkable. Due to climatic changes, the increasing import of dogs from endemic regions, the increase of tourism within Europe and the spatial expansion of potential vectors, it is recommended to protect all dogs in Germany prophylactically from vector-borne infections independent of origin or region. Because of the zoonotic potential of some pathogens, the prophylaxis, treatment and screening of vector-borne infections in dogs are also of great importance for human medicine [2].

## Conclusions

More than one third of dogs (35%) were positive for at least one pathogen. Dogs, which are imported from countries which are endemic for vector-borne infections should be thoroughly tested using direct and indirect detection methods. Furthermore, a second examination should be considered in recently imported dogs and infections with a long prepatency or a long time until seroconversion (e.g. *L. infantum* and *Dirofilaria* spp. after six months). The owners of imported dogs should be informed extensively about the diseases and their risks.

## Abbreviations

Ab: Antibody
Ag: Antigen
ELISA: Enzyme-linked immunosorbent assay
IFAT: Indirect immunofluorescence test
PCR: Polymerase chain reaction
TH1: T-helper-cells 1
